# Hype or hope? Ketamine for the treatment of depression: results from the application of deep learning to Twitter posts from 2010 to 2023

**DOI:** 10.3389/fpsyt.2024.1369727

**Published:** 2024-05-10

**Authors:** Qin Xiang Ng, Yu Liang Lim, Clarence Ong, Silas New, Johnson Fam, Tau Ming Liew

**Affiliations:** ^1^Health Services Research Unit, Singapore General Hospital, Singapore, Singapore; ^2^Saw Swee Hock School of Public Health, National University of Singapore and National University Health System, Singapore, Singapore; ^3^Department of General Medicine, Tan Tock Seng Hospital, Singapore, Singapore; ^4^Department of Pharmacy, Changi General Hospital, Singapore, Singapore; ^5^Department of Psychiatry, Singapore General Hospital, Singapore, Singapore; ^6^SingHealth Duke-NUS Medicine Academic Clinical Programme, Duke-NUS Medical School, Singapore, Singapore; ^7^Health Services and Systems Research, Duke-NUS Medical School, Singapore, Singapore

**Keywords:** ketamine, depression, anti-depressant, social media, BERT

## Abstract

**Objective:**

To investigate societal perceptions of ketamine’s use in depression therapy by analysing Twitter posts from January 1, 2010 to April 1, 2023.

**Methods:**

Using Twitter as the social media platform of choice, and employing search terms based on (depression OR depressed OR depressive) AND (ketamine OR esketamine OR Spravato), we collected English-language tweets from January 1, 2010, to April 1, 2023. Using unsupervised machine learning and natural language processing (NLP) techniques, including Bidirectional Encoder Representations from Transformers (BERT) and BERTopic, the study identified prevalent topics surrounding public chatter around the use of ketamine in depression treatment. Manual thematic analyses further refined these topics into themes.

**Results:**

Out of an initial dataset of 99,405 tweets, after removing duplicate tweets, re-tweets and tweets posted by organizations over Twitter, 18,899 unique tweets from presumably individual users were analysed. Analysis of temporal trends revealed a shift in public attitudes, particularly after the United States Food and Drug Administration (FDA)’s 2019 approval of ketamine for depression. Three major themes emerged: a changing regulatory landscape, cautious optimism, and personal experiences with the drug. There was an initial spike in discussions post-FDA approval in 2019. Thereafter, cautious optimism (Theme 2) decreased among the general public, with more personal accounts (Theme 3) highlighting the potential benefits for some treatment-resistant patients. Limitations of the study include Twitter’s inherent biases towards younger, English-speaking demographics.

**Conclusion:**

In summary, the public’s multifaceted perception leans towards a hopeful stance on ketamine’s therapeutic potential for depression.

## 1 Introduction

Depression is a primary driver of global disability, with significant public health implications ranging from work absenteeism to ballooning socioeconomic impacts relating to both direct and indirect healthcare expenses (1). Contemporary treatment strategies, which mostly target deficits in monoaminergic neurotransmission, grapple with two significant challenges: a protracted delay in therapeutic onset (spanning weeks to months) and a suboptimal response rate, with nearly one-third of patients failing multiple treatment trials and developing treatment-resistant depression (TRD) (2, 3).

For years, psychopharmacologic options have remained static since the debut of selective serotonin reuptake inhibitors (SSRIs) in the late 1980s, but the recent discovery of ketamine as a rapid anti-depressant has changed the narrative (4). Traditionally used as an anaesthetic, ketamine, an N-Methyl-D-Aspartate (NMDA) glutamate receptor antagonist, showed prompt antidepressant effects in several studies (done in the early and mid-2000s) involving patients with major depressive disorder (MDD) after just one subanaesthetic infusion (5, 6). A subsequent trial in 2010, recruiting both unipolar and bipolar depression patients (inclusive of those with TRD), have substantiated this finding, with some heralding a new paradigm in depression management (7). Ketamine’s complex mechanism of action likely involves myriad neurotransmitter systems, from opioidergic to muscarinic systems and beyond (4).

The United States Food and Drug Administration (US FDA) approved the use of Esketamine (Spravato), a specific enantiomer of racemic ketamine, for TRD in 2019 (8). Yet, while there is a burgeoning optimism about ketamine’s therapeutic potential, numerous uncertainties and reservations remain. Critics point to a lack of response maintenance and raise concerns over potential short and long-term side-effects, notably its capacity for misuse (9, 10).

Twitter, a social media behemoth boasting over 250 million global users, offers a rich platform for microblogging, with its tweets (capped at 280 characters) serving as invaluable data points for research and public sentiment analysis (11, 12). This study aims to uncover public opinions surrounding ketamine’s therapeutic use in depression, as represented by posts or tweets over Twitter. In today’s digital age, where opinions form and spread rapidly, gauging public perceptions is crucial (12). These opinions can potentially sway the direction of medical regulations and acceptance. The National Institute for Health and Care Excellence (NICE) in the United Kingdom also emphasise the importance of discussion and agreement on the treatment plan between the attending physician and the patient in their published guidelines (13); public discourse and opinions regarding ketamine as a treatment option could influence the patient’s choice or willingness to adhere to the chosen pharmacologic approach in addition to medical regulations. Hence, this understanding is pivotal for healthcare providers, regulators, patients, and the wider community. Twitter is also a suitable data source as individuals with mental health issues might tend to shy away from in-person interviews or focus groups but choose to openly discuss their mental health on social media platforms (14) or go to social media for information (15).

The study objectives extend beyond merely cataloguing public opinions on ketamine in depression therapy over the past decade. We also hope to offer insights that can guide subsequent research, clinical application, and health policy decisions. As popularity and interest in the use of ketamine grows in mental health discussions, a comprehensive understanding of societal perceptions becomes all the more invaluable.

## 2 Methods

### 2.1 Extraction of tweets

The methodology for the present study was adapted from previous infodemiology studies that also utilised Twitter to investigate public perceptions and manifested emotions on a particular topic (11, 12). Using Twitter as the social media platform of choice, and employing search terms based on (depression OR depressed OR depressive) AND (ketamine OR esketamine OR Spravato), we collected English-language tweets from January 1, 2010, to April 1, 2023. Extraction took place via Twitter’s Application Programming Interface (API) using an academic developer account, allowing the download of up to 10 million tweets per month without sampling. Retweets and duplicate tweets were excluded from analysis. The study had a global scope, with no restriction on tweet origin country. Retweets and duplicate tweets (i.e., tweets with identical sentences and words) were excluded from analysis. Tweets by organizations (e.g. agencies, news outlets and businesses) were also excluded from analysis.

### 2.2 Natural language processing

We utilized Bidirectional Encoder Representations from Transformers (BERT) to process the large volume of free-text data from Twitter. BERT is one of the state-of-the-art approaches in natural language processing, to understand the meaning of words and sentences in a way that is similar to how humans do, by taking into account the context in which they are used.

It was developed by Google in 2018 (16) and has since been deployed in Google search engine for all the English language search queries (17). Unlike previous language models, BERT is able to process text bidirectionally, i.e. it can take into account both the preceding and following words in a sentence when predicting the meaning of a given word. Moreover, BERT has been pre-trained on a large volume of text data in a Transformer-based neural network architecture, through the approach of masked language modelling which involves randomly masking some words in a sentence and training the model to predict the masked words based on their surrounding context (16). Named Entity Recognition (NER), which is a technique in BERT, was used to identify individual and organisational users based on the usernames of the tweets (18). BERT NER has been trained using a pre-training and fine-tuning approach, and it uses a sequence labelling approach, where the model takes a sequence of tokens (words or sub-words) as input and predicts a label for each token, indicating whether it belongs to an entity or not, and if so, what type of entity it is. It is able to recognise four types of entities: location (LOC), organisation (ORG), person (PER) and miscellaneous (MISC). In this study, we included tweets that were identified as persons (PER) through BERT. To elaborate, Twitter dataset has two separate columns (i.e. free text for tweets, and a separate column for user names), we specifically applied NER to the “user name” column, assuming that user accounts typically use names that reflect individual identities for personal accounts and organizational identities for non-personal accounts. Nevertheless, we do acknowledge the potential limitation where user names were not stated clearly or were misleading.

Thereafter, BERT-based Topic Modelling (19) was employed to generate interpretable key concerns on the public discussions surrounding the use of ketamine to treat depression. The BERTopic model utilized in this study is open-source (https://maartengr.github.io/BERTopic/index.html). Topic Modelling is an unsupervised machine learning technique that analyses words and phrases within text documents to extract hidden patterns and group similar concepts together to generate interpretable topics (20). It is akin to thematic analysis in traditional qualitative methodology but unlike thematic analysis, Topic Modelling does not require manual labour to group the text data and hence is well-suited for analyses of large volume of text data such as in this study. The steps involved in the BERTopic model process are illustrated in **Supplementary Figure S1**, and the number of topics generated is determined by topic coherence, with a Miscellaneous grouping where diverse or outlier content is aggregated.

### 2.3 Thematic analysis to further refine themes

Output from Topic Modelling was examined by the study investigators to ensure coherence of the identified topics. Descriptive label of each topic was manually crafted by the study investigators based on identified keywords and sample tweets of each topic. Then, the topics were further grouped into themes by the study investigators using the inductive and iterative processes of reflexive thematic analysis as introduced by Braun and Clarke (21). Thematic analysis provides a detailed and nuanced analysis, and is useful for studying individual opinions and viewpoints. By reading and re-reading the included tweets, the study investigators familiarized themselves with the data, produced preliminary codes, formulated overarching themes, reviewed and refined themes, defined and specified themes, and produced a write-up (21). Study investigators reviewed the tweets independently, and coding disagreements were resolved by further discussion until consensus was reached. The study investigators also moved back and forth between the different steps during the analysis in an iterative manner.

### 2.4 Ethical approval

Ethics approval for the study was granted by the SingHealth Centralised Institutional Review Board (CIRB) of Singapore (reference number: 2021/2717). This study did not directly involve human participants. All data used in the present study were collected in accordance with Twitter’s terms of use (22).

## 3 Results

### 3.1 Retrieval of tweets

**Figure 1:**
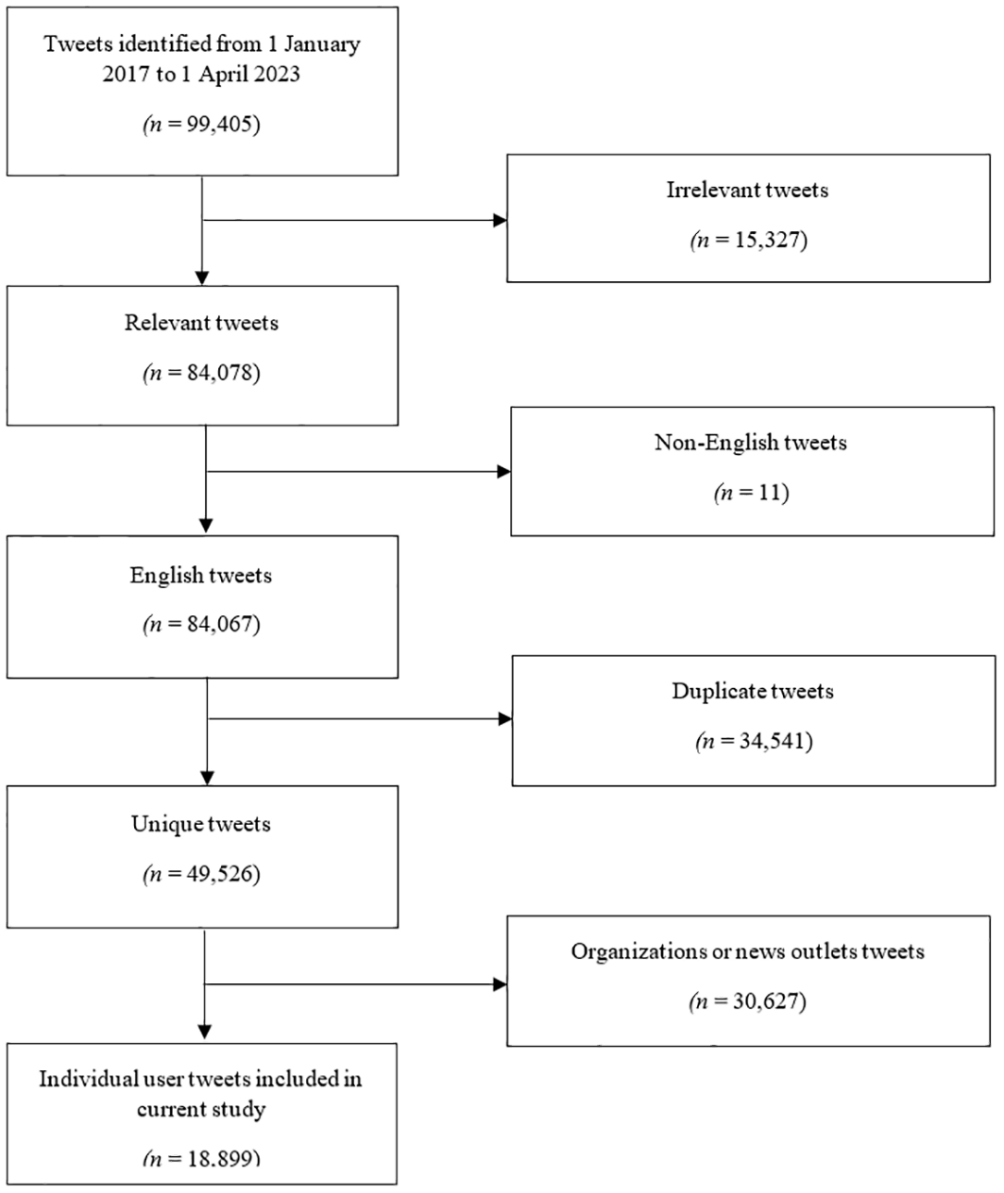
Flowchart illustrating the selection process for relevant tweets.

Out of an initial dataset of 99,405 tweets, a total of 49,526 unique English-language tweets were selected. Within this dataset, 30,627 tweets were identified as originating from organizations over Twitter, while 18,899 unique tweets from presumably individual users were chosen for analysis. **Figure 1** provides a visual representation of the tweet selection process.

**Figure 2:**
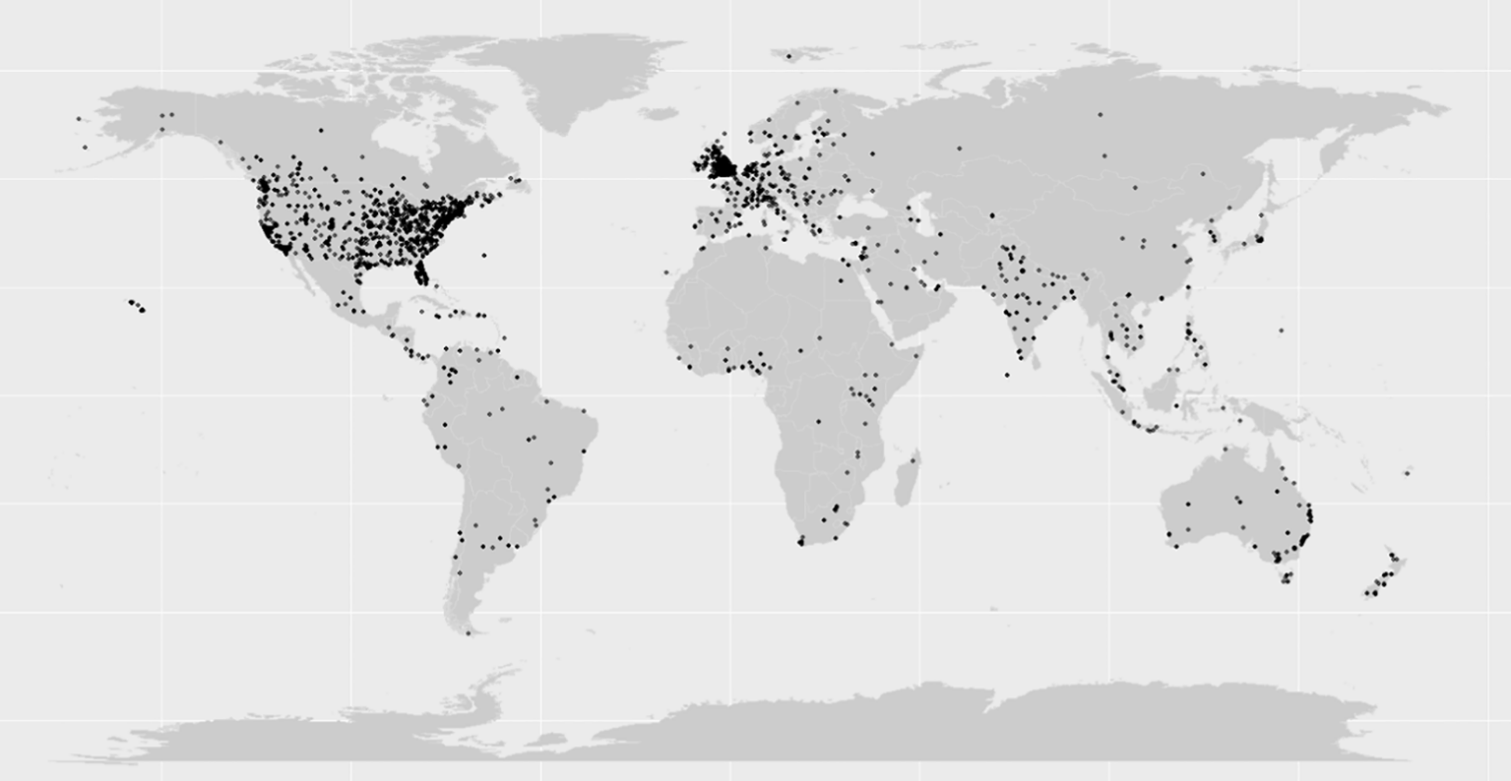
Geographical locations of the tweets included in this study (each tweet is indicated by a black dot on the map).

Analysis of the geolocation of the tweets found that the majority of these tweets originate from North America (51.9%) and Europe (13.1%), in alignment with the general demographic of English-speaking Twitter users (as shown in **Figure 2**) (23).

### 3.2 Topic modelling

A total of 12 topics were generated using the BERTopic model. The cumulative prevalence of these 12 topics amounted to 89.2%. The remaining 10.8% stemmed from a topic that was excluded from the present results, as the BERT NLP model created a Miscellaneous category to encompass all tweets that did not fit into any specific topic. These non-fitting tweets were identified as outliers by the NLP model, aiding in the reduction of noise that could potentially impact the representation of topics.

A significant majority of the tweets, specifically 12,842 tweets (68.0%), revolved around Topic 1. This topic primarily centered on the hope for a novel and efficacious treatment for depression.

### 3.3 Thematic analysis

Through qualitative thematic analysis, we organized the topics into three main themes. These themes are as follows: Theme 1: Changing Attitudes and Regulatory Landscape Regarding Ketamine’s Therapeutic Use (encompassing Topics 2, 5, 6, 7, 9 and 12), Theme 2: Cautious Optimism (comprising Topics 3, 4, 10, and 11), and Theme 3: Personal Experiences and Recommendations (comprising Topics 1 and 8). **Table 1** contains details regarding the individual topics, key words and sample tweets within each theme. A more detailed list of representative tweets from each topic is also available in the **Supplementary Material** (**Supplementary Table S1**) for further review. These themes, along with their sub-topics, are elaborated on below.

**Table 1:** Themes related to the public discussions on ketamine use for depression therapy, along with the respective topics and sample tweets (N = 18,899).

Topic label *(keywords)*	Sample tweets	Number of tweets, n (%)
Theme 1: Changing attitudes and regulation around the therapeutic use of ketamine			
Topic 2: Psychedelics as potential therapeutic agents (*psilocybin, psychedelics, psychedelic, mdma, ptsd, lsd, mushrooms, anxiety, therapy, mental*)	“There’s some really interesting research being conducted about therapeutic effects of microdosing hallucinogens like Ketamine, ‘shrooms (psilocybin) and LSD. Have been shown to help with depression, ptsd, and addiction” “Yep, and with the taboos wearing off milder psychedelics Like ketamine, mdma etc have also been used as treatment for depression, ptsd etc. this space is only going to get wilder/more interesting”	1009 (5.3)
Topic 5: Effects of ketamine on suicidal thoughts *(suicidal, suicidal thoughts, thoughts, suicide, ideation, suicidal ideation, depression suicidal, patients, suicidality, reduce)*	“Ketamine might be a life saver for suicidal depression. Long term use still a question. Nevertheless, watch the marketing in the US kick into gear. via #bipolar #bipolarspectrum #bipolaradvantage #depression #nami” “Ketamine Linked to Reduced Suicidal Thoughts, #Depression, #Anxiety #Psychiatry #Psychology #Child #AdolescentPsychiatry #MentalHealth #PsychiatryNursing #AddictionPsychiatry #Telepsychiatry #Anxiety #DepressionDisorder”	454 (2.4)
Topic 6: Repurposing a recreational drug for therapeutic purposes *(party, party drug, party drug ketamine, drug ketamine, closer, illegal party, illegal party drug, drug ketamine help, illegal, drug ketamine closer)*	“We’ll definitely party if it works: Ketamine’s journey from medicine to party drug back to medicine as a major breakthrough in depression treatment” “Party Drug’s Power To Fight Depression Puzzles Scientists #ketamine #SpecialK”	308 (1.6)
Topic 7: Ketamine as a treatment option for bipolar depression *(bipolar, bipolar depression, bipolar disorder, depression bipolar, minutes, ketamine bipolar, bipolar depression minutes, depression minutes, ketamine bipolar depression, improved bipolar depression)*	“Ketamine, the latest, greatest treatment for bipolar and depression” “IV Ketamine may be effective in treating bipolar depression in patients unresponsive to other treatments”	246 (1.3)
Topic 9: Announcement of FDA approval of esketamine nasal spray for depression *(nasal, esketamine nasal spray, esketamine nasal, nasal spray, spray, esketamine, approves esketamine nasal, approves esketamine, approves, fda approves esketamine)*	“Micro-dosing hallucinogens to treat depression. FDA Approves Esketamine Nasal Spray” “A nice example of pharmaceutics and formulation being used to address a pharmacological problem. Nasal administration should be harder to abuse than an injection. FDA Approves Esketamine Nasal Spray For Hard-To-Treat Depression”	173 (0.9)
Topic 12: Ketamine previous status as a street drug *(special, ketamine special, club, club drug, special ketamine, known, aka, drug special, aka special, street)*	“Special K aka Ketamine could help thousands with severe depression, doctors say” “The horse tranquilizer/club drug known as ketamine (Special K) has the potential to revolutionize depression treatment”	104 (0.6)
Theme 2: Cautious optimism			
Topic 3: Debate on esketamine for treatment of depression (*esketamine, spravato, esketamine treatment, esketamine treatment resistant, approval, esketamine depression, patients, intranasal, intranasal esketamine, depression esketamine*)	“Esketamine is hyped as superior to other drugs for treatment-resistant depression or as a therapy that can produce rapid results, two points studies don’t really support” “Nice thread on critiques of esketamine study against depression, one issue is placebo effect of meeting people for treatment was too high”	794 (4.2)
Topic 4: Reactions to FDA approval of a ketamine-based nasal spray for depression treatment *(nasal, spray, nasal spray, ketamine nasal, ketamine nasal spray, spray depression, nasal spray depression, approves, fda approves, ketamine like nasal)*	“FDA approves new ketamine-based treatment for depression. Special K in a nasal spray. What could go wrong? #publichealth” “FDA approved a new formulation of #ketamine, delivered as a nasal spray, for treatment-resistant #depression. Stops #glutamate from turning on the NMDA receptor that seems to be maxed out in several different #mentalhealth disorders.”	411 (2.2)
Topic 10: Skepticism on the use of a club drug for treatment *(ketamine, drug ketamine, depression club, depression club drug, club drug cure, drug cure, ketamine club drug, ketamine club)*	“Oh another rabbit hole, how will this turn out … special K -a club drug/illegal not so long ago. Why no studies on MMJ? Because it is still illegal- federally” “who would have though club drug Ketamine could have such a positive effect on depression”	161 (0.9)
Topic 11: Ketamine as a ‘horse tranquilizer’ *(horse, horse tranquilizer, tranquilizer, horses, ketamine horse, humans, ketamine horse tranquilizer, horse drug, horse tranquilizer used, tranquilizer used)*	“New studies have shown that the horse tranquiliser ketamine may mitigate depression! I find these old-fashioned narratives a soothing, even stultifying window on a solid and navigable world” “Now they want to give a vets former horse tranquillizer to those with depression! How about some peace, love and understanding? #ketamine”	144 (0.8)
Theme 3: Personal anecdotes and recommendations			
Topic 1: Hope for treatment-resistant depression *(therapy, anxiety, ketamine treatment, pain, ptsd, depressed, infusions, patients)*	“If you can find a doc to get ketamine treatment, try that. It really helps my husband who had treatment resistant depression” “#Ketamine infusion has been a lifesaver for many with suicidal depression.”	12,842 (68.0)
Topic 8: Comparing ketamine and electroconvulsive therapy for depression treatment *(ect, electroconvulsive therapy, electroconvulsive, therapy, ect ketamine, trial, ketamine ect, facebook, depressive)*	**“**My sister lost a huge chunk of her memories through ECT, albeit in the late 1970s. Surely we know enough now that Psilocybe and Ketamine should be mainstreamed especially for profoundly depressed and traumatised subjects” “- 1 naturalistic and 5 RCTs were included - A total of 340 patients (162 ECT; 178 Ketamine) - Patients had an either a DSM-5 or ICD-10 diangosis of depression - Primary assessed outcome was ‘improvement of depressive symptoms’ - All Ketamine patients were also ECT candidates”	197 (1.0)

#### 3.3.1 Theme 1: changing attitudes and regulation around the therapeutic use of ketamine

Theme 1 composed of six topics, accounting for 12.1% of tweets. A significant number of tweets, approximately 5.3% of the dataset, emphasized the potential therapeutic capabilities of psychedelics, including ketamine, psilocybin, and LSD, especially in the realm of depression and PTSD treatments (Topic 2). This changing perspective was further illustrated by discussions highlighting the effects of ketamine on suicidal thoughts, representing 2.4% of the tweets, underscoring the drug’s potential in providing rapid relief (Topic 5). There were also tweets illustrating the effectiveness of ketamine in bipolar depression (Topic 7). The transformation of ketamine’s image, from a recreational party drug to a promising therapeutic agent, was another topic of interest, accounting for 1.6% of the dataset (Topic 6). Paralleling FDA’s approval (Topic 9), the once-stigmatized narrative of ketamine as a “horse tranquilizer” also emerged, albeit in a smaller subset of tweets (0.8%), juxtaposing older perceptions with its newfound therapeutic potential.

#### 3.3.2 Theme 2: cautious optimism

This theme contained four topics and 8.1% of tweets. In general, topics here encapsulated the public’s guarded yet hopeful outlook. Within this theme, a debate on the use of esketamine for treating depression stood out, making up 4.2% of the discussions (Topic 3). Many users expressed concerns and critiques, questioning the efficacy of esketamine compared to other standard treatments. The FDA’s approval of a ketamine-based nasal spray for depression treatment sparked reactions that formed 2.2% of the tweet pool, with the public oscillating between hope and apprehension (Topic 4). Skepticism surrounding ketamine’s prior status as a club drug was evident in 0.9% of the tweets (Topic 10), and conversations invoking its association as a ‘horse tranquilizer’ made up another 0.8% (Topic 11), indicating lingering reservations amidst the optimism.

#### 3.3.3 Theme 3: personal anecdotes and recommendations

Theme 3, comprising two topics and 69.0% of tweets, was the most dominant theme. A total of 68% of the tweets revolved around the hope that ketamine offers for treatment-resistant depression (Topic 1). These tweets were replete with personal anecdotes, testimonials, and recommendations, painting a picture of desperation met with a glimmer of hope. A niche but notable discussion (1% of tweets) compared ketamine to electroconvulsive therapy (Topic 8), indicating a preference for the former for (likely treatment-resistant) depression due to fewer side effects and potential effectiveness.

### 3.4 Analysis of temporal trends

**Figure 3:**
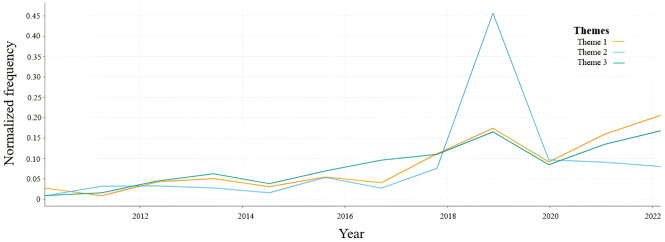
Temporal trends in the normalized frequency of tweets belonging to Themes 1, 2 and 3.

The temporal trends of these themes were also analysed in relation to the number of tweets posted for each topic between the years 2010 and 2023 (as shown in **Figure 3**). Observations revealed that discussions related to the utilization of ketamine experienced an uptick and peak in approximately 2019, which likely coincides with the FDA’s announcement of its approval of ketamine for depression therapy (US FDA, 2019). Subsequently, all three themes exhibited a decline around 2020, with a slight resurgence in tweets pertaining to Themes 1 (Changing Attitudes and Regulatory Landscape Regarding Ketamine’s Therapeutic Use) and 3 (Personal Experiences and Recommendations), while tweets associated with Theme 2 (Cautious Optimism) continued to decrease. This pattern may suggest a potential decline in cautious optimism regarding ketamine’s application in depression therapy, or reflect a natural decay in human attention following a surge in public interest.

## 4 Discussion

This study, through the exploration of public opinions expressed over Twitter on the therapeutic application of ketamine in depression, provides insight into the prevailing societal perceptions and the evolving landscape over the past six years, tracing its journey from an anaesthetic to a ‘party drug’, and now a novel antidepressant (5, 24). The significance of understanding public opinion on ketamine’s use in depression therapy (as presented in our analysis) is twofold. First, as regulatory bodies and healthcare providers contemplate the broader adoption of ketamine treatments, understanding public sentiment can guide educational and policy initiatives to address concerns and expectations. Second, patient advocacy groups and pharmaceutical entities can leverage these insights to better tailor communication and support services to address misconceptions and enhance patient engagement.

As reflected in our findings, there has been a notable shift in attitudes towards the therapeutic application of ketamine. As more regulators worldwide allow the use of ketamine-based treatments, the transition of ketamine into a potential novel treatment for depression has spurred public discourse and changes in public attitudes. The pivotal point seemed to be the US FDA’s approval in 2019, which was not only a validation for the clinical community but also catalysed a significant rise in public discourse around the topic. FDA’s approval was accompanied by an upsurge of tweets encapsulating cautious optimism, which mirrors the clinical community’s sentiments, where the initial enthusiasm meets the reality of clinical implementation, side effect profiles, and potential misuse (25, 26). Various questions and concerns exist in the public discourse and discussions that hint at an informed populace that is not easily swayed by hype and headlines. The decline observed post-2019 could be ascribed to the natural decay and waning of interest that commonly occurs following a peak in attention. Alternatively, it might signify the impact of recent clinical trials on ketamine, which further consolidated the real-world effectiveness of ketamine therapy for depressive symptoms (27, 28).

Notably, there was no apparent uptick in the number of tweets belonging to Theme 2 (cautious optimism) despite a second approval by the US FDA for the use of esketamine for suicidal patients with major depressive disorder in August 2020 (29). The growing evidence base (28) and approvals by FDA could have muted critics of ketamine therapy and sparked more hope than cautious optimism among the general public.

Personal narratives have always been a powerful medium to gauge the real-world impact of any intervention and Twitter has often been an avenue for individuals to search and share personal health questions and information (30). The discussions under Theme 3 offers valuable insights into patient experiences, and analysing the temporal trends for the various themes, there is a noticeable uptick in tweets falling under Themes 1 and 3, especially those recounting personal accounts of positive outcomes from ketamine therapy. This suggests that ketamine may indeed possess substantial antidepressant potential for certain individuals who otherwise exhibit resistance to conventional treatments. Notwithstanding various apprehensions, the observed variability in its effects is widely recognized and constitutes a focal point of ongoing research efforts (31).

Nonetheless, the primary limitation of this study is the inherent bias of Twitter as a platform. It may not be representative of the broader public sentiment, as it tends to skew towards younger, English-speaking demographics and might overlook non-English discussions on the topic (23). Further studies exploring sentiments across different social media platforms, languages, and cultures might provide a more holistic view of global perceptions. Second, as there is no predefined glossary of search terms for ketamine’s use as an antidepressant over Twitter, this means that certain sentiments may not have been picked up using our present search strategy and it may have introduced some selection bias. Third, without a reliable way to select and compare tweets from healthcare providers as opposed to non-healthcare providers, some of the themes and interpretations may not be representative of the lay public. As discussed earlier, we also acknowledge the potential limitation of using NER to identify individual user tweets and suggest that future studies might explore more sophisticated techniques or manual verification to increase the accuracy of tweet classification.

## 5 Conclusion

From the changing attitudes and increased acceptance of ketamine and other psychedelics for therapeutic purposes (Theme 1) to the cautious optimism, skepticism, and debates surrounding the drug (Theme 2), and personal anecdotes emphasizing its potential benefits (Theme 3), it is evident that public perceptions on this subject is multifaceted. Taken together, the narrative around ketamine is layered and nuanced. While there are still concerns and debates, the overarching sentiment from the general public seems to lean towards optimism, reflecting a growing hope in the potential of ketamine as a viable treatment for depression.

## Data availability statement

The original contributions presented in the study are included in the article/**Supplementary Material**. Further inquiries can be directed to the corresponding author.

## Ethics statement

The studies involving humans were approved by SingHealth Centralized Institutional Review Board (CIRB), Singapore. The studies were conducted in accordance with the local legislation and institutional requirements. Written informed consent for participation was not required from the participants or the participants’ legal guardians/next of kin in accordance with the national legislation and institutional requirements.

## Author contributions

QN: Conceptualization, Formal analysis, Investigation, Writing – original draft, Writing – review & editing. YL: Formal analysis, Investigation, Writing – original draft, Writing – review & editing. CO: Formal analysis, Investigation, Writing – original draft, Writing – review & editing. SN: Formal analysis, Investigation, Writing – original draft, Writing – review & editing. JF: Formal analysis, Writing – original draft, Writing – review & editing. TL: Conceptualization, Investigation, Methodology, Software, Supervision, Writing – original draft, Writing – review & editing.

## Supplementary material

Supplementary Figure S1 Workflow of the BERTopic model process. BERT, Bidirectional Encoder Representations from Transformers; HDBSCAN, Hierarchical Density-based Spatial Clustering of Applications with Noise; TF-IDF, Term Frequency–Inverse Document Frequency; UMAP, Uniform Manifold Approximation and Projection.
