# Supplementary material for: Hype or hope? Ketamine for the treatment of depression: results from the application of deep learning to Twitter posts from 2010 to 2023
Source: Front Psychiatry. 2024 May 10;15:1369727. doi: 10.3389/fpsyt.2024.1369727 (PMC11117142; doi:10.3389/fpsyt.2024.1369727)
Supplement: Supplementary file 1 [file Table_1.docx]

Supplementary Material

# Supplementary Data

**Table S1.** Sample tweets for the various topics, as per BERTopic modelling

| **S/N** | **Tweet** |
| --- | --- |
| Topic 1: Hope for treatment-resistant depression | |
|  | 'In biggest advance for #depression in years, FDA approves novel treatment' "The medicine has a complex legacy because it is a component of ketamine, which was approved years ago as an anesthetic and was once popular as a party drug called Special K..." |
|  | These results are promising and support larger #studies to identify novel interventions to promote lasting #recovery #eatingdisorders #anorexia #keto #diet #depression #ketamine |
|  | #Ketamine infusion has been a lifesaver for many with suicidal depression. |
|  | If you can find a doc to get ketamine treatment, try that. It really helps my husband who had treatment resistant depression |
|  | Meanwhile F.D.A. expert panel recommends that the active ingredients of ketamine be approved as a treatment for depression |
|  | FDA approved ketamine treatment for my life stealing depression treatment. |
|  | S KETAMINE is going be available for depression, but it will only be given at a Dr office and you will have to be watched 2 hr. This will be given after you failed 2 other treatments for Depression. What do you think about this? |
|  | Meanwhile F.D.A. expert panel recommends that the active ingredients of ketamine be approved as a treatment for depression |
|  | A ketamine-like drug is the first new antidepressant to get FDA approval in years as FDA becomes increasingly concerned about the growth rates of depression. FDA has also granted breakthrough therapy designation to psilocybin in late 2018. |
|  | Hey can you offer any evidence that the FDA changed its stance on IV ketamine & has now approved it for depression after that statement you sent to membership yesterday? Because IV is still OFF LABEL for depression. #inaccurate |
| Topic 2: Psychedelics as potential therapeutic agents | |
|  | There's some really interesting research being conducted about therapeutic effects of microdosing hallucinogens like Ketamine, 'shrooms (psilocybin) and LSD. Have been shown to help with depression, ptsd, and addiction. |
|  | WORLD IN 2023: PSYCHEDELIC DRUGS AS PRESCRIPTION MEDICINE 3 Drugs in Phase III Trials. 1. MDMA+Psychotherapy for PTSD 2. Psilocybin (from Mushroom) for Depression 3. Ketamine (used in nightclubs) based therapy for alcohol use disorder |
|  | My province is the first in Canada to regulate the use of psychedelics to treat depression. I have only had access to ketamine but I believe that public health care providers (covered by the government) will now make these other drugs available in a similar manner. |
|  | Exactly. LSD and ketamine too have showed enormous promise in the treatment of anxiety and depression, as has psilocybin (magic mushies). |
|  | Different psychedelics seem better at treating certain disorders than others. For example: ketamine for depression, MDMA for CPTSD, and ibogaine for opioid addiction |
|  | Surprisingly not far off. Ketamine and Psilocybin mushrooms are both being looked at as serious remedies for depression. Who would have ever thought you could solve an issue WITHOUT pills? Oh yeah, everyone in the last few thousand years. |
|  | Taking halucinogens every now and then is like a reset. It keeps my misanthropy at bay. If they succeed in legalizing psilocybin and LSD for depression, it will revolutionize mental healthcare. They have legalized ketamine for depression, and that's good. |
|  | Cannabis, ketamine, and Lsd have all proven to be more effective treatments of depression. Too bad we communicate with each other like kids. |
|  | About time: micodosing psilocybin and MMDA has been helping PTSD and depression in clinical trials in Canada for years, but are illegal; ketamine, a legal alterntative is making headway in treating depression at a new Toronto clinic. |
|  | Ketamine is already being used to treat chronic pain and depression. MDMA therapy is going to be approved for therapeutic use in a couple years. Psilocybin mushrooms are going through FDA-approved research to be a treatment for PTSD, depression, and OCD. |
| Topic 3: Debate on esketamine for treatment of depression | |
|  | Esketamine should be Available for Treatment Resistant Depression, estimates 2.7 Million People in the UK have TRD #PsychedelicMedicine #ParadigmShift #TRD #MedicalSupervision #PatientVoice |
|  | F.D.A. Panel Recommends New Depression Treatment. Esketamine has been reported to provide rapid results as compared to our existing antidepressants in the market. |
|  | Important article Esketamine for treatment resistant depression: a trick of smoke and mirrors? |
|  | So I got a call today from UC Health about beginning Esketamine treatment. This is an intranasal treatment for treatment-resistant depression. Since the ECT treatment proved to do nothing for the depression and anxiety, my psychiatrist talked to the ECT doctor at UC and put |
|  | #Esketamine For Resistant Depression .. Needs Monitoring for 2 Hrs post administration |
|  | Nice thread on critiques of esKetamine study against depression, one issue is placebo effect of meeting people for treatment was too high. |
|  | the problem is, they see a big improvement from baseline. The fact is, this is 80% placebo effect , amply demonstrated by the recent esketamine approval, where even with "treatment resistant" depressed patients, placebo was 80% as good. |
|  | You may get your wish. Treatment resistant depression is exactly what esketamine is designed for. |
|  | Short-term results cannot provide a rational basis for treatment recommendations for depression... It is not enough to simply render the patients acutely euphoric with a hallucinogenic substance |
|  | Im a little confused...how does regression to the mean explain the vastly different rate; timelines of improvement seen in placebo groups in studies of SSRIs, adjuncts eg antipsychotics, esketamine & psychedelics in depression? |
| Topic 4: Reactions to FDA approval of a ketamine-based nasal spray for depression treatment | |
|  | Hopefully there is some good legit science backing this before it's rushed to market. A Ketamine-Based Nasal Spray for Depression Could Soon be Approved by the FDA |
|  | Apparently we can spray ketamine up our noses to cure depression? Yeah sure dude why not |
|  | FDA approves new ketamine-based treatment for depression. Special K in a nasal spray. What could go wrong? #publichealth |
|  | Ketamine Nasal Spray Expected To Be Approved For Treatment Of Depression. How abuseable is this going to be? |
|  | OMG did you know they have ketamine nasal sprays for treatment -resistant depression? This is so cool! Before Kid I just thought Ketamine was something gym bros did to bulk up. The world of science is vast and amazing! |
|  | do you have any thoughts about the horrible horse tranqilizer ketamine proposed to be used as a nasal spray for severely depressed people? I have seen it used clinically. It is abominable!!! |
|  | On Tuesday (March 5), the U.S. Food and Drug Administration (FDA) approved a ketamine-like nasal spray for patients with depression who haven't responded to other treatments. But what makes... |
|  | I live with treatment-resistant depression every freaking day, I'm hopeful that this could halp me. FDA could approve ketamine nasal spray as depression treatment |
|  | Watching the news...talking about a ketamine nose spray for depression.... I'm done. The world is over. Medicine is being destroyed by idiots. |
|  | A major stepping stone towards treatment of major depressive disorders. A Ketamine-Based Nasal Spray for Depression Could Soon be Approved by the FDA via |
| Topic 5: Effects of ketamine on suicidal thoughts | |
|  | Just out-- in a review across ketamine clinical trials, suicidal ideation was more responsive to the placebo effect as compared to other depressive symptoms such as depression or anhedonia. Congrats to Bartholt and the all-star list of co-authors! |
|  | Depressed People Who Express Suicidal Thoughts Find Relief In IV Ketamine Treatment |
|  | Ketamine might be a life saver for suicidal depression. Long term use still a question. Nevertheless, watch the marketing in the US kick into gear. via #bipolar #bipolarspectrum #bipolaradvantage #depression #nami |
|  | Adjunctive Ketamine Appears to Reduce Suicidal Thoughts in Depressed Patients for Up to Six Weeks #feedly |
|  | Ketamine can be extremely effective for those with treatment resistant depression and suicidal thoughts. |
|  | New double blind RCT finds no evidence ketamine treats suicidality or depression. Important finding given the possibly over-eager adoption of this drug in clincial practice on the basis of scant evidence eg |
|  | #Ketamine for acute treatment of severe suicidal ideation: d. bld, ran'ised placebo-controlled trial (France) Really useful, comments on this article too #mentalhealth #depression #mentalillness #NMPPU #SOMNMPCPD #TeamPatient #MedEd #clinicaleducation |
|  | Why it's worth considering adding ketamine to the treatment regimen of people with severe depression or suicidal tendencies, writes |
|  | Anesthetic Ketamine May Reduce Suicidal Thoughts In Depressed People Suicide is among the world's top three... |
|  | Ketamine Linked to Reduced Suicidal Thoughts, #Depression, #Anxiety #Psychiatry #Psychology #Child #AdolescentPsychiatry #MentalHealth #PsychiatryNursing #AddictionPsychiatry #Telepsychiatry #Anxiety #DepressionDisorder |
| Topic 6: Repurposing a recreational drug for therapeutic purposes | |
|  | The Party Drug Ketamine Is Becoming An Unlikely Treatment For Depression via |
|  | Party drug Ketamine could be used to treat #depression by doctors in the UK by the end of the year. |
|  | Scientists are excited about ketamine, a party drug that could prevent depression |
|  | Depression Therapy With Party-Drug Roots Faces FDA Panel Review #Healthcare #Depression #FDA #Review #Ketamine |
|  | Ketamine could cure severe depression - UK researchers think the tranquiliser party drug could treat |
|  | #NewsTechnology - Party drug Ketamine may help treat severe depression |
|  | Party Drug Ketamine Being Tested to Treat Depression |
|  | 'Party drug' and animal tranquilizer Ketamine for depression? |
|  | Party drug ketamine closer to approval for depression |
|  | Ketamine, Onetime Party Drug, May Be 'Miracle' Cure for Severe Depression |
| Topic 7: Ketamine as a treatment option for bipolar depression | |
|  | Interesting: lithium does not beat placebo for prolonging the acute antidepressant effects of ketamine in unipolar depression |
|  | Bipolar-Depression Improvement Follows Ketamine Infusion |
|  | NeuroRx boasts big about a new ketamine combo for bipolar depression |
|  | Did you know, in a study, 71% of patients who took ketamine injection experienced improvements in their symptoms of depression? Researchers also saw potential for ketamine in addressing symptoms of bipolar disorder. |
|  | Huge congrats on this important paper demonstrating the real world effectiveness and safety of IV #ketamine for treatment resistant bipolar depression FYI: Now doing 2 trials to further evaluate efficacy in BD |
|  | Blood Test Predicts Which Bipolar Patients Will Respond to Ketamine: Two-thirds of bipolar... #Bipolar #Depression |
|  | Our data on the largest sample to date demonstrating positive Safety and tolerability of IV #ketamine in adults with major #depressive or #bipolardisorder: results from the Canadian rapid treatment center of excellence |
|  | Psychiatry therapeutics has experimented a big progress during the last 20 and specially 10 years, which the pessimistic Dr. Allen's statement doesn't seem to consider. There is still much work to do for treating bipolar depression, I hope Ketamine derived meds may help us. |
|  | fascinated by this: Can Ketamine Help Treat Depression in Bipolar Patients? |
|  | Ketamine produces rapid antidepressant effects in patients with treatment-resistant bipolar depression |
| Topic 8: Comparing ketamine and electroconvulsive therapy for depression treatment | |
|  | Thanks! The ECT unfortunately didn't work for me. It is usually highly successful, but I didn't benefit much. What ended up beating the depression was small doses of Ketamine, which has been newly FDA approved as a treatment for depression |
|  | Given #ketamine has shown efficacy in treating severe depression, should it be the anaesthetic for #ECT (instead of propofol) - no evidence it boosts effectiveness of ECT. |
|  | #ECT Ketamine for depression: where do we go from here? |
|  | If you get the genetic testing and esketamine isn’t likely to work (happened to me), ECT is also shown to be very effective for treatment resistant depression and is not as scary as it seems!! |
|  | - 1 naturalistic and 5 RCTs were included - A total of 340 patients (162 ECT; 178 Ketamine) - Patients had an either a DSM-5 or ICD-10 diangosis of depression - Primary assessed outcome was 'improvement of depressive symptoms' - All Ketamine patients were also ECT candidates |
|  | Medscape: ECT is more effective than IV ketamine for patients experiencing a major depressive episode in new findings that are in line with the KetECT study the first head-to-head trial of ketamine and ECT. |
|  | My sister lost a huge chunk of her memories through ECT, albeit in the late 1970s. Surely we know enough now that Psilocybe and Ketamine should be mainstreamed especially for profoundly depressed and traumatised subjects |
|  | Ketamine is as effective as ECT in improving depressive symptoms With some important caveats |
|  | Also evidence shows that Ketamine reduces depression in those who don't respond to ECT |
|  | Article fr Some pts unresponsive 2 depression treatments may benefit fr ketamine. An alternative to ECT? |
| Topic 9: Announcement of FDA approval of esketamine nasal spray for depression | |
|  | The FDA approved a new treatment for depression. Esketamine is a nasal spray drug used to fight intractable depression within hours. |
|  | But OMG the price. I'm daily amazed that more US citizens don't flee to Canada. FDA Approves Esketamine Nasal Spray For Hard-To-Treat Depression |
|  | Micro-dosing hallucinogens to treat depression. FDA Approves Esketamine Nasal Spray |
|  | Approves Esketamine Nasal Spray For Hard-To-Treat Depression #depression |
|  | The Food and Drug Administration ( has approved the sale of #esketamine, an anti-depression nasal spray, purported to be effective within 24 hours. via |
|  | New post: FDA approves esketamine nasal spray, the first new major depression drug in more than 30 years |
|  | Medical News Today: The FDA approve esketamine nasal spray for severe depression |
|  | This article is making headlines Esketamine nasal spray, approved as an adjunctive treatment for treatment-resistant #depression in adults, may carry a clear potential for serious adverse events. Psychotherapy and Psychosomatics |
|  | (ASPIRE I) Study: #Esketamine nasal spray rapidly reduced #depression symptoms in adult patients with MDD who had moderate to severe depression and suicidal ideation with intent. |
|  | FDA Committees Vote on Esketamine Nasal Spray for Treatment-Resistant Depression This may help many with severe #depression |
| Topic 10: Skepticism on the use of a club drug for treatment | |
|  | *Special K* is so 90s/early 2000's. Nowadays heroin/fentanyl, xanax, adderall, all qualify as *club drugs* too. *Ketamine* is still used to treat pain, depression, inflamation, etc. |
|  | Laura Knowles The Club Drug Cure Ketamine Treats Depression |
|  | reading from A Mystery Partly Solved: How the 'Club Drug' Ketamine Lifts Depression So Quickly |
|  | Get on one if ur fed up! 'club drug' ketamine to treat depression could be an important advancement |
|  | Ketamine-Like Drugs Treat Depression Fast with Minimal Side Effects \| weird that a "club drug" could help w/depression |
|  | Interesting read in the on using ketamine, a club drug, to treat depression. |
|  | Using club drug ketamine to treat depression could be an important advancement \| via cc |
|  | Oh another rabbit hole, how will this turn out... special K -a club drug/illegal not so long ago. Why no studies on MMJ? Because it is still illegal- federally. How Ketamine Treats Depression: It Activates Opioid Receptors, Study Finds |
|  | How 'club drug' ketamine lifts depression so quickly: |
|  | After trying everything for anxiety... Club Drug Ketamine Gains Traction As A Treatment For Depression |
| Topic 11: Ketamine as a ‘horse tranquilizer’ | |
|  | Getting tired of this comment from the media. Ketamine, a horse tranquilizer, is now used to treat human depression. Only ignorant people can't grasp how medicine can be used in both animals and humans. |
|  | Ketamine, a horse tranquilizer, is used for bipolar and depression in humans. It is not approved for that use. |
|  | Would they sneer at someone taking ketamine for depression as taking "horse drugs"? |
|  | New studies have shown that the horse tranquiliser ketamine may mitigate depression! I find these old-fashioned narratives a soothing, even stultifying window on a solid and navigable world. |
|  | I know memes are fun but ketamine was not invented to be a horse tranquilizer. Horses are just incredibly weak structurally so its the best sedative for the animal. in 5 years when you can buy ket at a CVS for your basic depression, youll laugh at these memes even more |
|  | I've never watched the show but im wondering if they ever address why he doesn't just do ketamine to treat his depression. probably super easy to get prescribed as a horse |
|  | Ketamine really to help fight #depression Holy Jesus that stuff can put one of my horses or a 2200 pound bull on their sides in minutes!! #NotForEveryone |
|  | Ketamine,which is commonly used as a horse tranquilizer, is showing promise as a treatment for depression:Users report UNBRIDLED happiness! |
|  | Horse drug ketamine is remarkable for depressed via no wonder all my mates are such happy bastards *cough* |
|  | Amazing that treating depression can go from just two SSRIs straight to horse tranquilizers. Prozac? No. Zoloft? Meh. Ketamine? Lets see what happens! |
| Topic 12: Ketamine previous status as a street drug | |
|  | For years, I've been following the progress of ketamine (special K) as a depression treatment. Huge upside potential for some patients: |
|  | Andy Vermaut shares:Americans Are Using Ketamine To Treat Depression With Lack Of Government Regulation: Famous in party circles, the drug is called "Special K," and it is known to produce an out-of-body hallucinogenic high. Thank you. |
|  | Doctors Are Testing Ketamine (Special K) as an Instant Depression Remedy |
|  | Ketamine can help to cure depression and I thought special k was just high in fiber |
|  | #health Ketamine, or "Special K," effectively treats severe depression in study - CBS News |
|  | Best depression pun of the day: Ketamine, a psychoactive party drug better known as Special K, has pharmaceutical companies riding high. |
|  | Special K aka Ketamine could help thousands with severe depression, doctors say |
|  | Is Club Drug 'Special K' a Quick Fix for Depression?: New studies show that ketamine, an anesthetic |
|  | 'Special K' #Ketamine May Ward Off #Depression, #Bipolar Disorder : Health |
|  | TIL: Ketamine, also known as "Special K" cures depression in one dose. |
