# Supplementary figures and images for: Hype or hope? Ketamine for the treatment of depression: results from the application of deep learning to Twitter posts from 2010 to 2023
Source: Front Psychiatry. 2024 May 10;15:1369727. doi: 10.3389/fpsyt.2024.1369727 (PMC11117142; doi:10.3389/fpsyt.2024.1369727)

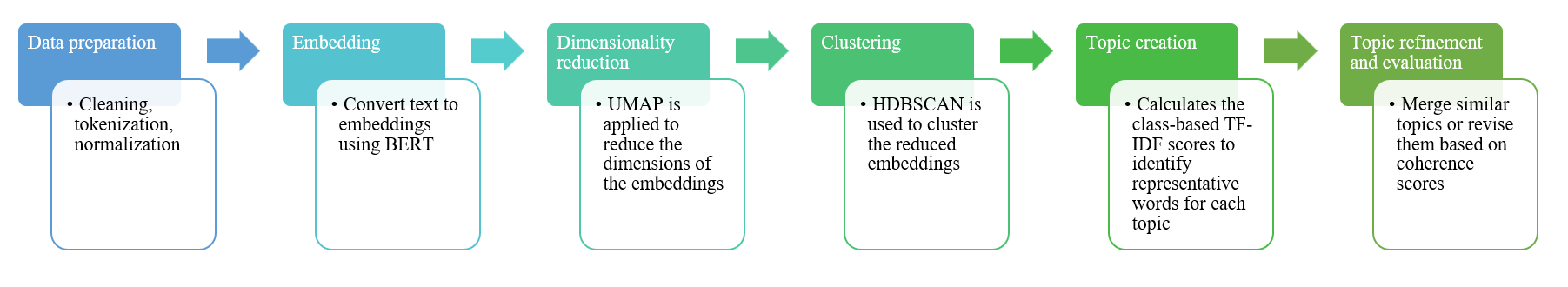

Supplement: Supplementary Figure S1 — Workflow of the BERTopic model process. BERT, Bidirectional Encoder Representations from Transformers; HDBSCAN, Hierarchical Density-based Spatial Clustering of Applications with Noise; TF-IDF, Term Frequency–Inverse Document Frequency; UMAP, Uniform Manifold Approximation and Projection. [file Image_1.tif]
